# Transcatheter arterial and venous embolisation with α-hexyl cyanoacrylate MagicGlue®: short-term safety and efficacy outcomes

**DOI:** 10.1186/s42155-025-00535-0

**Published:** 2025-03-20

**Authors:** Hamza Sawalha, Olivier Chevallier, Mohamed Fouad, Taninokuchi Tomassini Makoto, Comby Pierre-Olivier, Ludwig Serge Aho-Glele, Romaric Loffroy

**Affiliations:** 1 Department of Vascular and Interventional Radiology, Image-Guided Therapy Center, François-Mitterrand University Hospital, 14 Rue Paul Gaffarel, BP 77908, 21000 Dijon, France; 2 ICMUB Laboratory, UMR CNRS 6302, University of Burgundy, 9 Avenue Alain Savary, 210000 Dijon, France; 3 Department of Emergency Radiology and Neuroradiology, François-Mitterrand University Hospital, 14 Rue Paul Gaffarel, BP 77908, 21000 Dijon, France; 4 Department of Epidemiology, Statistics and Clinical Research, François-Mitterrand University Hospital, 14 Rue Paul Gaffarel, BP 77908, 21000 Dijon, France

**Keywords:** Embolisation, Artery, Vein, Cyanoacrylate, Outcomes

## Abstract

**Purpose:**

Our purpose was to assess the feasibility and the short-term safety and efficacy outcomes of a wide range of transcatheter arterial and venous embolisation procedures done using α-hexyl-cyanoacrylate (AHCA)-MagicGlue® in patients with bleeding and non-bleeding disorders.

**Methods:**

This single-centre retrospective study included consecutive patients who underwent emergent or planned AHCA-MagicGlue® transcatheter embolisation between February 2019 and September 2023. Technical success, clinical success, 30-day mortality, and complication rates were evaluated.

**Results:**

We included 101 patients with a mean age of 49.9 ± 20.5 years who underwent arterial (*n* = 43, 42.6%) or venous (*n* = 58, 57.4%) embolisation for bleeding (*n* = 16, 15.8%) or other reasons (*n* = 85, 84.2%). The technical success rate was 100%. After a mean follow-up of 2.2 months, the clinical success rate was 94% in patients with bleeding and 95% in other patients; 1 patient died of multi-organ failure unrelated to the procedure. In the 22 patients with prostatic artery embolisation, statistically significant improvements were recorded at 3 months versus baseline for the International Prostate Symptoms Score (IPSS) (10.0 ± 5.8 vs. 20.8 ± 7.3, *p* = 0.001), IPSS quality-of-life score (2.0 ± 1.4 vs. 5.0 ± 1.0; *p* = 0.001), and prostate volume (67.8 ± 38.0 mL vs. 96.7 ± 47.4 mL, *p* = 0.001). Adverse events occurred in 11 (10.9%) patients and were major in 4 and minor in 7 patients.

**Conclusions:**

MagicGlue® transcatheter arterial and venous embolisation is feasible, effective, and safe for bleeding and non-bleeding conditions across a broad range of anatomic sites.

## Introduction

Interventional radiology has emerged as a pivotal component of the management of vascular lesions, as it is both minimally invasive and effective. Among transcatheter embolisation techniques, the injection of synthetic adhesives such as cyanoacrylates has garnered considerable interest based on strong evidence of effectiveness and versatility [1–6].

Among cyanoacrylates, N-butyl cyanoacrylate (NBCA) is the most widely used [2, 4, 5]. Histoacryl® (B/Braun, Tuttlingen, Germany) and Trufill® (Cordis, Miami Lakes, FL) are the oldest NBCAs but have proven difficult to use because of their fast polymerisation time [7]. In Glubran®2 (GEM Srl, Viareggio, Italy), NBCA is combined with the co-monomer metacryloxysulfolane to produce a more pliable and stable polymer whose milder exothermic reaction (45 °C instead of 90 °C) results in less inflammation and histotoxicity and whose slower polymerisation rate improves ease of use and decreases the risk of catheter entrapment [1, 7].

The new long-chain cyanoacrylate variant α-hexyl cyanoacrylate (AHCA)-MagicGlue® (Balt, Montmorency, France) has received limited attention for peripheral interventional radiology procedures but has demonstrated effectiveness in animal studies and for neurovascular interventions [8–10]. Compared to other glues, the greater number of methyl radicals in AHCA-MagicGlue® results in less cytotoxicity, adhesive strength, and pro-inflammatory potential, thereby increasing ease of use [10]. No large study has reported the use of AHCA-MagicGlue® as the main liquid embolic agent at peripheral and visceral sites.

The primary objective of this retrospective single-centre observational cohort study was to evaluate the feasibility and the short-term safety and efficacy outcomes of transcatheter AHCA-MagicGlue® embolisation used to treat bleeding and non-bleeding lesions at arterial and venous sites.

## Materials and methods

Neither submission to an ethics committee for approval nor collection of patient informed consent to study inclusion were required, in compliance with French law on retrospective assessments of de-identified health data. The study complied with the Declaration of Helsinki as amended in 2013. All patients provided informed consent to the embolisation procedure and were informed of the use of AHCA-MagicGlue®.

### Study design

We retrospectively reviewed the medical records of all patients who underwent emergent or planned transcatheter embolisation with AHCA-MagicGlue® for bleeding or non-bleeding lesions at any visceral or peripheral vascular site, between February 2019 and September 2023, at our university hospital. Patients were identified by searching the database maintained prospectively by our interventional radiology department. Patients with head or neck embolisation were not eligible for the study.

### Data collection

A single investigator retrieved the data from the patient files and recorded them on standardised forms. For each patient, baseline data were age, sex, type of procedure, vessel to be embolised, use of other embolics, Lipiodol® dilution ratio, events and follow-up duration. In patients who had prostatic artery embolisation (PAE), the International Prostate Symptoms Score (IPSS), IPSS quality-of-life score (IPSS-QoL), and prostate volume were also collected. For varicocele, the presence of pain and/or infertility, unilateral or bilateral topography, and grade were recorded. Finally, liver volume was evaluated in patients who underwent portal vein embolisation.

### Transcatheter embolisation technique

All angiography procedures were performed by the same experienced interventional radiologist using a Philips Allura Xper FD 20 angio room (Philips, Best, The Netherlands) and standard percutaneous transfemoral or transjugular catheterisation with a 5-Fr or 6-Fr sheath. The target vein or artery was selectively opacified via standard 4-Fr or 5-Fr catheters, and superselective arteriography was then performed using a 2.0- to 2.7-Fr co-axial microcatheter (Progreat; Terumo, Leuven, Belgium).

AHCA-MagicGlue® was used for all embolisation procedures. When the angiography was normal, the feeding vessel was embolised based on endoscopic or computed tomography (CT) findings. The microcatheter was introduced co-axially into the angiography catheter and advanced as distally as possible to avoid reflux of the embolic material into non-target vessels. AHCA-MagicGlue® was mixed with ethiodised oil (Lipiodol® Ultra Fluide; Guerbet, Roissy, France) in a ratio of 1:1 to 1:8 depending on the site and operator preference, as previously described [2, 6, 7]. After microcatheter flushing with 5% dextrose solution to avoid glue polymerisation within the lumen, the glue-Lipiodol® mixture was carefully injected under fluoroscopic monitoring. The ratio, volume, and injection rate of the mixture were at the discretion of the interventional radiologist, who based decisions on the size and flow of the embolised vessels. To avoid catheter-tip adhesion to the vessel wall, the microcatheter was quickly removed after the injection. Immediately after embolisation, angiography was performed to confirm vessel occlusion.

**Fig. 1:**
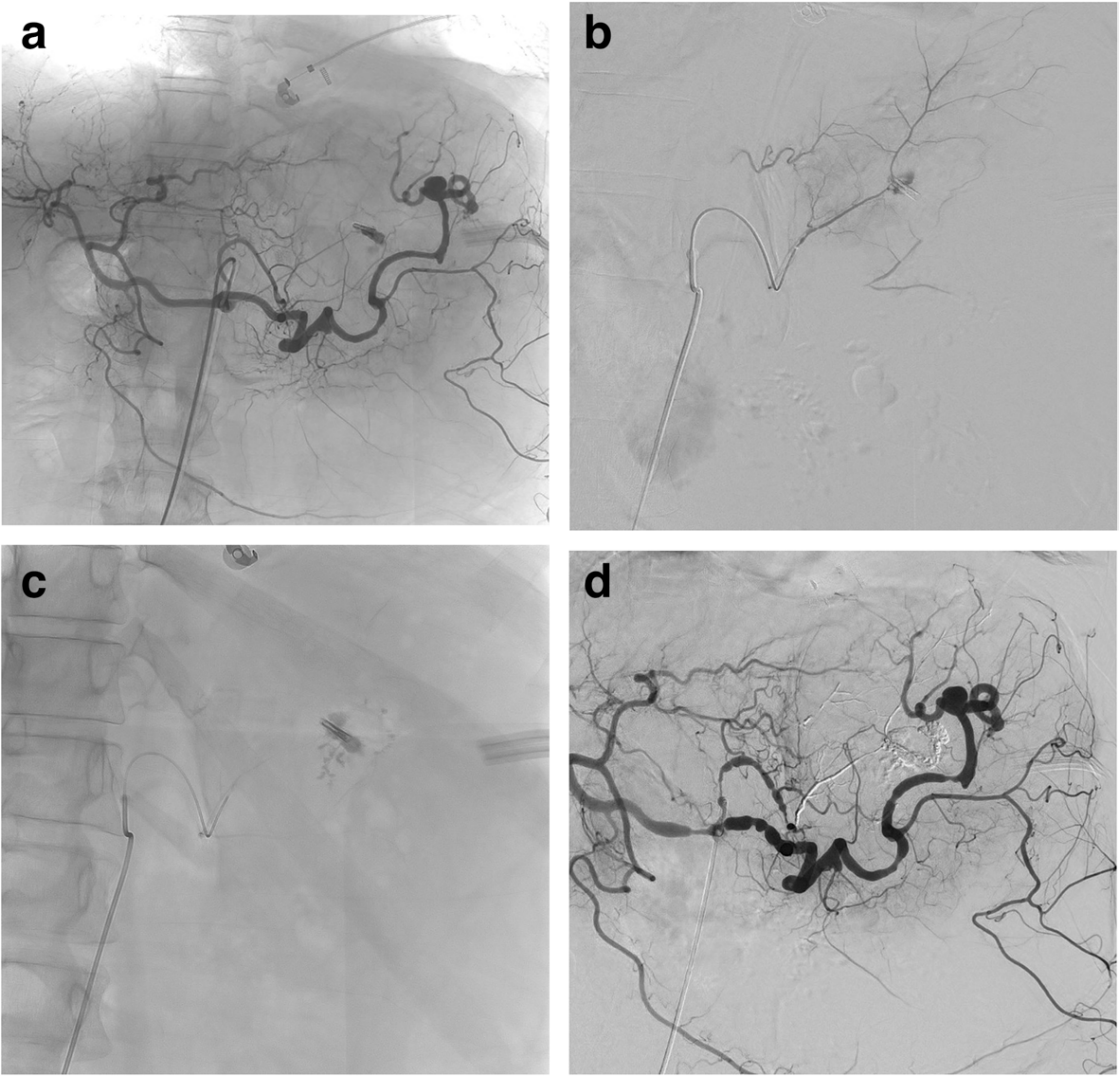
Acute upper gastrointestinal bleeding from a gastric peptic ulcer in a 65-year-old man with haemodynamic instability and impaired coagulation. **a** Angiography of the celiac trunk showing contrast medium extravasation from a small distal branch at the lesser curvature of the stomach, supplied by the left gastric artery. **b** Superselective angiogram of the feeding artery, guided by the presence of an endoscopic metallic clip, confirming active bleeding. **c** After microcatheterisation, embolisation with an AHCA-MagicGlue®/Lipiodol® mixture in a 1:3 ratio was successful in controlling the bleeding. **d** Final angiogram demonstrating successful occlusion of the bleeding artery with preserved patency of the collaterals. No re-bleeding occurred in this patient

In patients with bleeding from a visceral vessel, embolisation was performed as distally as possible. For upper gastrointestinal (GI) bleeding, embolisation was performed either empirically or based on angiography findings (Fig. 1), whereas for the lower GI only angiographically visible lesions were embolised. Embolisation was usually achieved using AHCA-MagicGlue® alone, in a 1:2 to 1:4 glue/Lipiodol® ratio or AHCA-MagicGlue® combined with other embolic agents, as previously described, after failure of endoscopic treatment (Fig. 1) [2, 3, 11]. For rectus sheath haematoma, the target vessel was the ipsilateral inferior epigastric artery. For psoas haematoma, the bleeding lumbar artery and the lumbar arteries immediately above and below it were embolised routinely, on both sides, to avoid revascularisation [12]. A single microcatheter was used, with liberal 5%-dextrose-solution flushing after removal from each previously embolised lumbar artery.

**Fig. 2:**
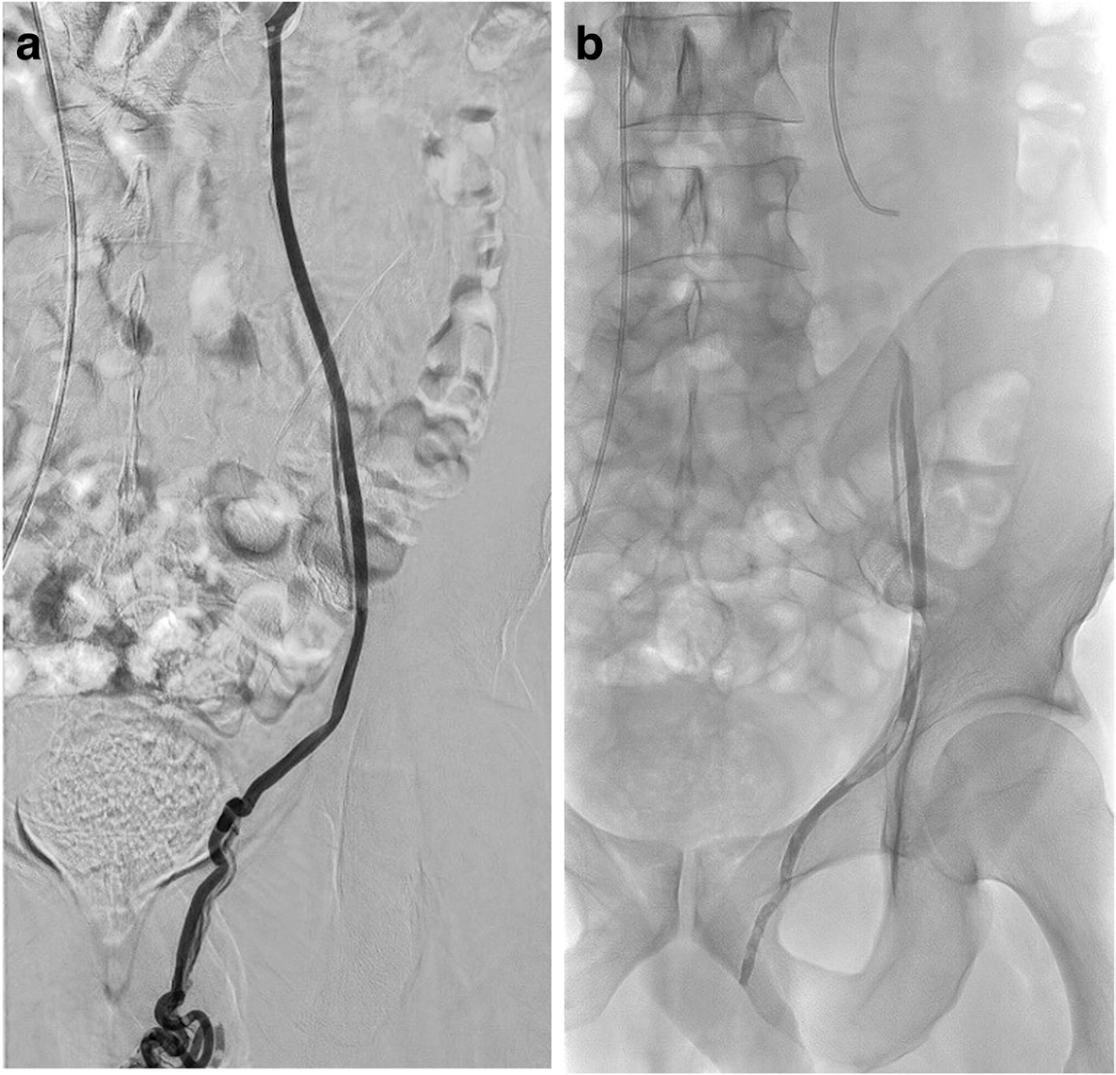
Left varicocele responsible for pain in a 28-year-old man. **a** Left spermatic venography through a 5-French Cobra catheter by the transvenous femoral approach showing two venous channels. **b** Plain radiograph after left spermatic vein embolisation using AHCA-MagicGlue®/Lipiodol® in a 1:1 ratio: note the glue cast along the main left spermatic vein with reflux into the second, smaller channel producing complete occlusion of all collaterals. The left testicle pain resolved within 1 month and the follow-up sonogram showed no reflux into the pampiniform plexus

For varicocele, embolisation sought to occlude the spermatic vein and the parallel and segmental veins, to cover the origins of all relevant side branches or renospermatic bypasses. A 5-Fr standard catheter was used to catheterise the left renal vein then the orifice of the left gonadal vein. A pre-embolisation venogram was then obtained during a Valsalva manoeuvre. A 2.7-Fr microcatheter (Progreat®, Terumo Interventional Systems, Japan) was introduced into the gonadal vein. Venograms were obtained at various levels while advancing the microcatheter from the orifice of the gonadal vein to the level of the pubic symphysis. The paraspermatic veins connecting collaterals or renospermatic bypasses to the internal spermatic vein were mapped. An AHCA-MagicGlue®/Lipiodol® ratio of 1:1 was used to achieve rapid polymerisation and avoid migration in the event of reflux. The glue-Lipiodol® mixture was injected under strict fluoroscopic control, with continuous manual injection and a display of real-time distribution [13]. The injection began in the distal intrapelvic segment of the gonadal vein, and the microcatheter was then withdrawn while the glue was injected under fluoroscopy guidance (Fig. 2). The injection was stopped before the pampiniform plexus was reached [13].

**Fig. 3:**
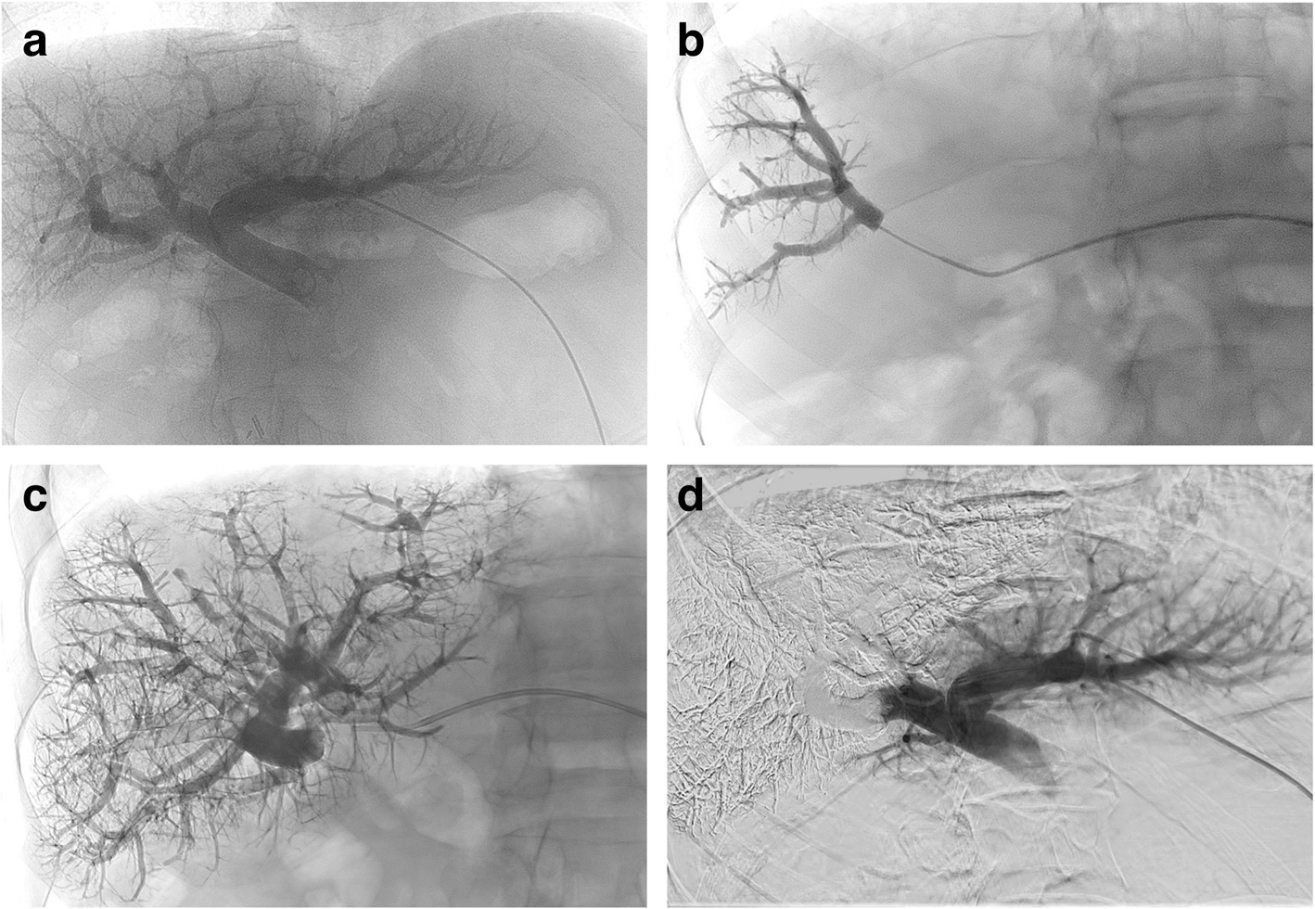
Right portal vein glue embolisation before surgery in a 56-year-old woman with hepatocellular carcinoma. **a** Portal venography with a 4-Fr standard catheter inserted through a 5-Fr sheath via the contralateral approach: note the normal subsegmental portal branches. **b** Start of selective glue embolisation of right portal vein branches through a 2.7-Fr microcatheter. **c** Radiograph after full embolisation of the right portal vein and its branches with AHCA-MagicGlue®/Lipiodol® in a 1:7 ratio showing the distribution of the radiopaque embolisation material. **d** Final portography revealing complete occlusion of the right portal vein branches and preserved blood flow in the left portal branches and segment IV veins. Hypertrophy of the left liver remnant occurred and right hepatectomy done 6 weeks after embolisation was uneventful

Portal vein embolisation usually consisted in total right portal vein embolisation through a contralateral approach, using a 1:7 glue/Lipiodol® ratio. All branches were filled by reflux during microcatheter withdrawal (Fig. 3).

**Fig. 4:**
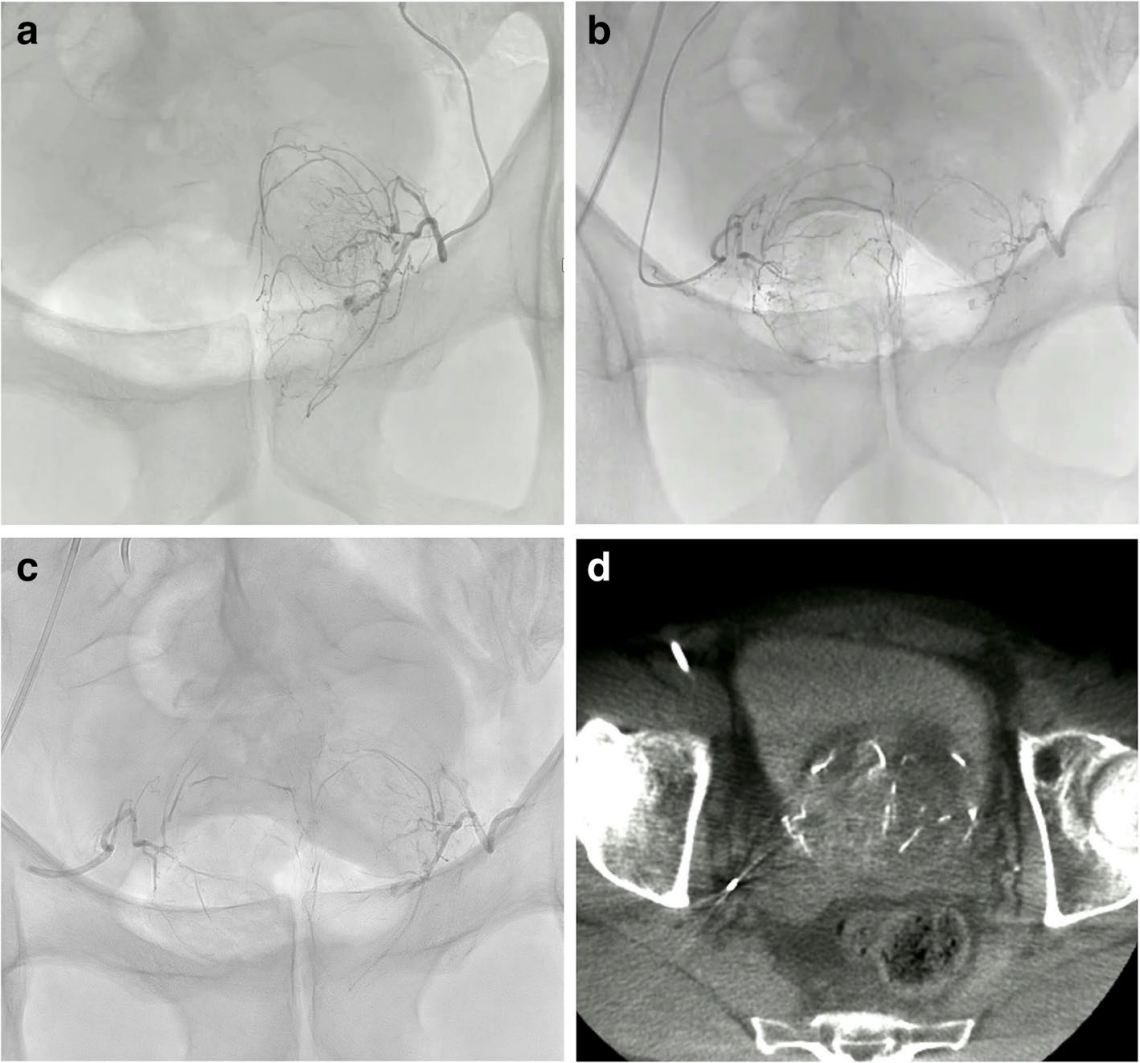
Seventy one-year-old man referred for prostatic artery embolisation (PAE) to treat lower urinary tract symptoms (LUTS) due to benign prostatic hyperplasia. **a** Selective left prostatic artery angiography with a 2.4-Fr microcatheter before PAE showing left prostatic lobe enlargement with a normal distal vascular bed. **b** Selective right prostatic artery angiography after PAE of the left prostatic artery with AHCA-MagicGlue®/Lipiodol® in a 1:8 ratio visualising the blood supply to the right prostatic lobe and demonstrating complete occlusion of both the distal and the proximal segments of the left prostatic artery. **c** Control angiogram after PAE of the right prostatic artery using the same mixture: note the cast in the distal and proximal branches of the right prostatic artery, with complete occlusion. **d** Axial cone-beam computed tomography with contrast injection after bilateral PAE: Lipiodol® uptake by both prostatic lobes with distal and proximal distribution of the AHCA-MagicGlue®/Lipiodol.® casts. Major improvements in the LUTS were noted 3 months after PAE compared to baseline (International Prostate Symptoms Score (IPSS), 4 vs. 21; IPSS-Quality of Life, 2 vs. 6; prostate volume, 59 vs. 91 mL)

For PAE, a microcatheter was introduced into the feeding artery on each side successively. Flushing of the microcatheters and vascular bed was achieved by injecting 10 mL of 5% dextrose on each side. A 1:8 AHCA-MagicGlue®/Lipiodol® ratio (0.5 mL of glue and 4 mL of lipiodol) was chosen to produce a fluid mixture allowing distal penetration, as previously described [6, 7, 14]. Free-flow or blocked-flow conditions were used depending on the size of the arteries. The injection was continued under continuous fluoroscopic monitoring until substantial reflux occurred and deep penetration was visible (Fig. 4). When deemed appropriate by the interventional radiologist, the “plug and push” technique was used to achieve deeper penetration, as previously described [14, 15]. The goal was distal embolisation and proximal occlusion of the prostatic arteries without occlusion of any collaterals. When appropriate, several arteries were embolised on the same side and/or, via anastomoses, on the contralateral side. Once the goal was achieved on one side, the microcatheter was immediately withdrawn and flushed with 5% dextrose then re-used on the other side if its condition allowed. At the end of the procedure, the catheter and sheath were removed and the femoral-artery puncture site was sealed by implanting an Exoseal™ (Cordis, Hialeah, FL) or FemoSeal™ (Terumo, Tokyo, Japan) device.

In patients with bleeding lesions, all embolic agents were released near the bleeding site until the angiography no longer showed extravasation and/or fluoroscopy showed occlusion of the target vessel.

### Outcomes

The arteriogram was considered positive if contrast-medium extravasation or a false aneurysm-like lesion was visible. Technical success was defined as complete target-vessel occlusion or, in patients with bleeding, cessation of extravasation.

The primary outcome was clinical success. In patients with bleeding, clinical success was defined as absence of persistent bleeding or re-bleeding requiring re-intervention within 30 days. Persistent bleeding was defined as continuing bleeding after embolisation, a greater than 2.0 g/dL decrease in the haemoglobin level, and/or lack of effectiveness of conservative medical treatment. Re-bleeding was defined as a new bleeding episode at the same site more than 48 h and less than 30 days after the procedure, defined using the same criteria. The definition for PAE was an IPSS-score decrease of at least 25% and 2 points combined with an IPSS-QoL score lower than 4/6 at follow-up. For varicocele, clinical success was absence of reflux by Duplex ultrasound (DUS) and absence of pain. Clinical success in patients with venous insufficiency at other sites was satisfactory imaging control and resolution of pain. Prophylactic arterial embolisation and embolisation of arterio-venous malformations was deemed clinically successful when imaging control was satisfactory. Finally, for portal vein embolisation, clinical success was defined as an at least 30% increase in left hepatic volume after 4–6 weeks.

Adverse events at any time during the first 30 post-procedural days were reported according to the standards of the Society of Interventional Radiology (SIR) as major complications if they required surgery and/or prolonged hospitalisation and as minor complications otherwise [16].

### Statistical analysis

Continuous variables were described as mean ± standard deviation (SD) with the 95% confidence interval if normally distributed and as median [25th–75th centile] otherwise [17]. Qualitative variables were described as n (%).

In the group with prostatic artery embolisation, the baseline and post-embolisation mean values of the IPSS, IPSS-QoL, and prostate volume were compared by applying the parametric paired Student *t*-test.

The statistical analyses were performed using STATA software version 18.0 (STATA, College Station, TX).

## Results

### Patients

We included 101 patients, whose main features are listed in Table 1. Table 2 reports the embolisation sites, glue/Lipiodol® ratios, and use of other embolic agents. AHCA-MagicGlue® was used alone in most patients.

**Table 1:** Baseline characteristics of the 101 study patients

Characteristics	Data
**Age, years**	
**Overall**	
Mean ± SD (range)	49.9 ± 20.5 (12 − 92)
Median [IQR]	55.0 [31.0–68.0]
**Haemostatic embolisation**	
Mean ± SD (range)	65.8 ± 7.2 (40 − 92)
Median [IQR]	67.0 [57.0–72.0]
**Prostatic artery embolisation**	
Mean ± SD (range)	68.2 ± 7.2 (55 − 81)
Median [IQR]	68.5 [65.5–73.5]
**Varicocele embolisation**	
Mean ± SD (range)	33.1 ± 12.8 (12 − 70)
Median [IQR]	31.0 [24–38.5]
**Other non-haemostatic embolisation**^**a**^	
Mean ± SD (range)	57.3 ± 19.8 (27 − 88)
Median [IQR]	59 [41.5–72.0]
**Sex, n (%)**	
**Overall**	
Males	92 (91.1)
Females	9 (8.9)
**Haemostatic embolisation**	
Males	13 (76.5)
Females	4 (23.5)
**Non-haemostatic embolisation**	
Males	79 (94.0)
Females	5 (6.0)

^a^prophylactic arterial embolisation (*n* = 7), other venous incontinence (*n* = 4), arteriovenous malformation (*n* = 2), and portal vein embolisation (*n* = 2)

**Table 2:** Technical features of the embolisation procedures

Variables	n (%)
**Type of vessel**	
Artery	43 (42.6)
Prostatic	22 (21.8)
Gastro-intestinal	6 (5.9)
Renal	6 (5.9)
Other	9 (8.9)
Vein	58 (57.4)
Spermatic	47 (46.5)
Pelvic/ovarian	4 (4.0)
Oesophageal	3 (3.0)
Portal	2 (2.0)
Other	2 (2.0
**AHCA-MagicGlue®/Lipiodol® ratio**	
1/1	54 (53.5)
1/3	4 (4.0)
1/4	14 (13.9)
1/5	6 (5.9)
1/7	1 (1.0)
1/8	22 (21.8)
**Embolic agent, n (%)**	
AHCA-MagicGlue® alone	93 (92.1)
AHCA-MagicGlue® combined with other embolics	8 (7.9)
* Coils*	*5 (4.9)*
* Gelatispon*	*2 (2.0)*
* Aetoxysclerol*	*1 (1.0)*

### Efficacy outcomes

Technical success was achieved in all patients. Follow-up data were missing for 1 patient. At a mean follow-up of 2.2 months, the clinical success rate in the remaining 100 patients was 95% (Table 3). Clinical success rates were 94% in the 17 patients with haemostatic procedures and 95% in the 83 other patients.

**Table 3:** Safety and efficacy outcomes

Variables	Data
**Technical success**^**a**^**, n (%)**	101 (100.0)
**Clinical success**^**b**^**, n (%)**	
Overall	95 (95.0)
Arterial haemostasis	13 (93.0)
Venous haemostasis	3 (100.0)
Prophylactic arterial embolisation	7 (85.0)
Prostatic artery embolisation	20 (95.0)
Varicocele causing pain	21 (91.0)
Varicocele causing infertility	24 (100.0)
Other venous incontinence	4 (100.0)
Arteriovenous malformation	2 (100.0)
Portal vein embolisation	2 (100.0)
**Complications according to SIR, n (%)**	
Total	11 (10.9)
* Minor*	*7 (6.9)*
* A*	*6 (5.9)*
* B*	*1 (1.0)*
* Major*	*4 (4.0)*
**30-day mortality**	1 (1)
**Follow-up (months)**	
Overall	
Mean ± SD	2.2 ± 1.5
Median [IQR]	2.0 [1.5–2.6]
Prostatic artery embolisation	
Mean ± SD	3.1 ± 0.8
Median [IQR]	3.0 [3.0–3.1]
Varicocele	
Mean ± SD	2.0 ± 0.3
Median [IQR]	2.0 [1.9–2.1]
Haemostatic embolisation	
Mean ± SD	1.0 ± 0.2
Median [IQR]	1.0 [1.0–1.0]

^a^defined as complete obliteration of the vessel documented by angiography at the end of the procedure

^b^see text for the definition, which varied according to the indication

*SIR* Society of Interventional Radiology

In the PAE group (Table 4), univariate analyses demonstrated significant improvements from baseline to 3 months, with absolute decreases of 10.8 points for the IPSS (*p* < 0.001), 2.0 points for the IPSS-QoL (*p* < 0.001), and 28.0 mL for prostate volume (*p* < 0.001).

**Table 4:** Prostatic artery embolisation group: efficacy outcomes at 3 months

Variables	Baseline	3 months	Absolute variation	*P* value
**IPSS**				
Mean ± SD	20.8 ± 7.3	10.0 ± 5.8	−10.8 (51.9%)	< 0.001
Median [IQR]	20.5 [16.0–26.0)	9.0 [6.0–13.0)	−11.5 (56.1%)	
**IPSS-QoL**				
Mean ± SD	5.0 ± 1.0	2.0 ± 1.4	−3.0 (60.0%)	< 0.001
Median [IQR]	5.0 [1.0–4.0)	2.0 [1.0–3.0)	−3.0 (60.0%)	
**Prostate volume, mL**				
Mean ± SD	96.7 ± 47.4	68.7 ± 38.0	−28.0 (39.0%)	< 0.001
Median [IQR]	80.0 [60.0–111.0)	59.0 [44.5–88.5)	−21.0 (26.2%)	

*IPSS* International Prostatic Symptoms Score, *IPSS-QoL* IPSS-associated quality-of life score

### Safety outcomes

Major adverse events occurred in 4 (4.0%) patients. None was attributable to the embolisation procedure. Infection requiring admission for less than 48 h developed in 2 patients, bleeding requiring re-intervention in 1 patient, and multiorgan failure with a fatal outcome on day 10 in 1 patient.

Minor adverse events developed in 7 (6.9%) patients and were all related to embolisation. Moderate post-PAE syndrome that resolved with conservative medical treatment occurred in 3 patients, asymptomatic and clinically irrelevant glue-cast migration in 2 patients, puncture-site haematoma in 1 patient, and superficial vein thrombosis in 1 patient.

## Discussion

Our findings in 101 patients support the safety and efficacy of AHCA-MagicGlue® embolisation across a broad range of indications and vascular sites. The clinical success rate was 95%. The few major complications were unrelated to the embolisation procedure. A single patient died within 30 days, from multi-organ failure unrelated to the embolisation procedure. Minor complications were rare and had no adverse clinical impact. No ischaemic complications occurred.

Transcatheter arterial and venous embolisation is now considered the reference standard treatment in a variety of situations based on feasibility, minimal invasiveness, and efficacy [2, 11–14]. Each of the many commercially available embolic materials has advantages and disadvantages [7]. The embolic material is usually chosen on a case-by-case basis, depending on various factors such as vascular anatomy and pathology and personal preference of the interventional radiologist. Cyanoacrylates have been less often used for peripheral embolisation compared to metallic coils and particulates. Recently, however, cyanoacrylate embolisation has been evaluated in an increasing number of studies and used in an expanding range of indications [1–6, 9, 14, 18–20].

An advantage of liquid adhesive embolics is low viscosity allowing easy injection through small or tortuous vessels [7, 20]. Cyanoacrylates have a high adhesive strength and polymerise in the presence of water, triggering an exothermic reaction that contributes to destroy the vascular endothelium. Few cyanoacrylates for endovascular use are commercially available worldwide. Among them, the NBCAs Trufill® and Histoacryl® have similar properties [7, 20, 21]. Trufill® is approved by the Federal Drug Administration (FDA) but is available only in the United States. Histoacryl® has been used for decades but has neither FDA approval nor the CE mark for endovascular embolisation. Disadvantages include the high polymerisation temperature of 90 °C and fast polymerisation within less than < 30 s The widely used mixture of NBCA and metacryloxysulfolane Glubran®2 has a polymerisation temperature of only 45 °C that produces less inflammation and histotoxicity than Histoacryl® and Trufill® [7, 21]. With AHCA-MagicGlue®, the slower polymerisation compared to Glubran®2 increases ease of use and decreases the risk of catheter entrapment, and the weaker exothermic reaction results in less pain during injection [8–10]. AHCA-MagicGlue® is CE marked for endovascular use. Mixing with Lipiodol® to ensure radio-opacity allows modulation of the polymerisation rate, which decreases as the proportion of Lipiodol® increases. Thus, with less Lipiodol®, occlusion is achieved faster, which may be desirable in patients with bleeding [14] but requires a short injection time and prompt catheter removal; while more Lipiodol® allows greater distality of penetration beyond the catheter tip [8–10, 14]. Similar to NBCA, AHCA-MagicGlue® remains effective in patients with coagulopathy [7].

In patients with varicocele, the few available studies showed very high success rates with glue embolisation [5, 13, 22–24]. Major advantages of glue are the persistence of vascular occlusion over time and penetration of the glue into the collaterals of the main gonadal vein. Glue migration into the pulmonary circulation can be avoided by using a 1:1 glue/Lipiodol® ratio to ensure prompt polymerisation. The glue should be injected slowly but decisively to avoid reflux with occlusion of non-target vessels [13, 23].

PAE was performed in a fifth of our patients, to treat incapacitating lower-urinary-tract symptoms due to benign prostatic hyperplasia. The IPSS, IPSS-QoL, and prostate volume were significantly improved after 3 months. No patient developed urinary incontinence, ejaculation dysfunction, or other major complications and none required re-admission. The procedure was done on an outpatient basis in all patients. Glue has several advantages over the microparticles used in many studies [25–28]. The shorter procedure duration translates into a lower radiation dose to the patient [14, 29]. The rapid glue polymerisation in the surface-to-core direction leaves no time for pre-existing vascular anastomoses to open and, therefore, decreases the risk of non-target embolisation [29, 30]. The Lipiodol® mixed with the glue provides better radiological visibility compared to particles. Glue injection under blocked-flow conditions allows the embolisation of arteries in which a microcatheter cannot be advanced, a common situation with low-flow or small prostatic arteries [15]. Compared to the free-flow technique, blocked-flow injection allows greater distality of embolisation, and the blocked catheter prevents glue reflux. Compared to NBCA, AHCA-MagicGlue® polymerises more slowly, decreasing the risk of catheter entrapment. The combined distal and proximal arterial occlusion achieved with glue embolisation may be associated with lower risks of long-term recanalisation and symptom recurrence compared to microparticles, although this point requires further investigation.

The limitations of our study include the retrospective data collection and small sample size. The recruitment at a single university-hospital centre and performance of all procedures by the same operator with considerable experience in glue embolisation may limit the general applicability of our findings. Furthermore, the choice of glue as the embolic agent was at the discretion of the operator, and selection bias may therefore have occurred. However, this point reflects daily clinical practice. The patients were heterogeneous, given our objective of assessing outcomes in a broad range of indications and vascular sites. Finally, only short-term outcome data were recorded. Prospective studies in larger samples, with a longer follow-up and comparisons of different embolic agents including cyanoacrylates should be performed.

## Conclusions

Our findings support the efficacy and safety of AHCA-MagicGlue® for transcatheter arterial and venous embolisation in various indications and at several anatomical sites. AHCA-MagicGlue® may be particularly useful in patients with coagulopathy and when the vessels are too narrow to allow distal embolisation by microcoils. Its ease of use and good safety profile may warrant widespread use by interventional radiologists. Randomised trials with longer follow-ups are warranted to compare embolic agents in various indications.

## Data Availability

Upon request.
